# Factors Affecting the Intention to Quit among Women Smokers in Turkey

**DOI:** 10.31557/APJCP.2020.21.11.3309

**Published:** 2020-11

**Authors:** Omer Alkan, Aysenur Demir

**Affiliations:** *Department of Econometrics, Faculty of Economics and Administrative Sciences, Ataturk University, Erzurum, Turkey. *

**Keywords:** Intention to quit, smoking cessation, multinomial probit regression, Global Adult Tobacco Survey, Turkey

## Abstract

**Objectives:**

Tobacco use is an important public health problem that affects adversely the quality of life. A person’s attitude toward quitting tobacco use can be reflected by the desire or intention to quit smoking. The aim of this study was to determine risk factors affecting women’s intention to quit tobacco in Turkey.

**Methods:**

In this study, the data obtained from the Global Adult Tobacco Survey (GATS) were used. The GATS is a standard method used in countries to monitor and evaluate the frequency of tobacco use in adults and tobacco control practices. The data used in the study was obtained from the GATS carried out in Turkey in 2008 and 2012. The data related to 1248 women smoking tobacco were used in the analysis. The relationship between women’s intention to quit tobacco use and socio-demographic and economic variables was examined.

**Results:**

Men were excluded from the analysis because the focus of the study was women. It was determined that 732 of 1248 women using tobacco intend to quit smoking tobacco. 40.4% of women smoking tobacco are primary school graduates. Women, who were aware of anti-smoking messages and exposed to stimulants that promote smoking, were 36.4% and 27% more likely to intend to stop tobacco use after the next month, respectively. It was observed that women living in a house, where smoking is allowed, are less likely to quit smoking than others (ME = -0.522). This shows the importance of domestic restrictions.

**Conclusion:**

Intending to quit is an important preliminary step to quit. Understanding the factors associated with the intention to quit smoking can help tobacco users to stop using it and shape effective policies to increase the quit rates.

## Introduction

Nowadays public health is considered to be one of the most important indicators of life quality. Public health is one of the fundamental issues t++o be emphasized in all countries (Pascual Sáez et al., 2015). Tobacco use, which has been reported to cause a 30-fold increase in cancer rates and a 9-fold increase in the risk of heart attack, is a risk factor for chronic obstructive pulmonary disease (COPD) and pharyngeal, esophageal, bladder, laryngeal and pancreatic cancers (Yang et al., 2012). Tobacco use is one of the most preventable causes of death, and quitting tobacco use at an early age can reduce the mortality and morbidity resulting from its use (Doll et al., 2004). 

According to the World Health Organization report, the tobacco users account for 1.4 billion people across the world. Nowadays women constitute approximately 20% of more than 1 billion tobacco users in the World (WHO, 2019). Recent studies have indicated that sex differences in tobacco use disappeared, and tobacco companies targeted women aggressively in developing countries (Ganatra et al., 2007). In developed countries, tobacco consumption in women has increased paradoxically in the last century (WHO, 2010a). Women were preferred more in the advertising industry, and on the basis of this situation, the main aim was to emphasize the beauty, dignity, and freedom of women. By being drawn attention to these features, smoking has also increased due to women’s economic freedom (WHO, 2010b). Physiological, psychological and social factors, including the addictive effect of nicotine, have a role in the women’s continuation of smoking. Women are more vulnerable to the effects of physically used substances, so they are more prone to addiction. In addition, various studies have shown that women quit smoking more difficult than men, and when quitting smoking, they are more vulnerable to nerve, stress, depression, loss of weight control and negative withdrawal effects (Poole and Dell, 2005; WHO, 2010a). 

The cigarette is the most common way of consuming tobacco. Most smokers start smoking during adolescence and continue to be regular users during adulthood. Regardless of the age of quitting smoking, smoking cessation reduces the burden of tobacco-related diseases and increases the life expectancy of smokers (Nguyen et al., 2018). However, the greatest effects of smoking cessation are generally seen in those with a more short-term history of smoking (Nguyen et al., 2018). Furthermore, quitting smoking before age 30 can reduce most of the tobacco-related deaths (Horn et al., 2018). A person’s attitude toward quitting smoking can be reflected by the desire or intention to quit smoking (Põld and Pärna, 2018). The intention is an indication that a person is ready to exhibit a behavior (Ajzen, 1991). Motivating the individual’s intention to quit smoking, understanding the intention-related factors, and designing relevant psychosocial interventions may help smokers to quit smoking. The intention is an important determinant of smoking cessation (Grimshaw and Stanton, 2006). The United States Department of Health and Human Services (USDHHS) Clinical Practice Guideline for Treating Tobacco Use and Dependence recommends that the smoking cessation intervention should begin by assessing the user’s intention to quit. Having the intention of quitting smoking is strongly associated with previous attempts to quit smoking. Therefore, it is seen as an important preliminary step to quit smoking (Yang et al., 2012).

Smoking in women can result in diminished bone density and reproductive risks such as the increased likelihood of infertility, pregnancy complications, premature birth and stillbirth, in addition to smoking-related diseases in men (Manaf and Shamsuddin, 2008). Although it is known that women’s rates to quit smoking were lower than men, smoking was addressed by men rather than women in most societies (Perkins and Scott, 2008). Women’s tobacco consumption is not more evident than men’s tobacco consumption, and due to these socio-cultural characteristics, there is less access to quit smoking situation of women tobacco users and generalizable data collection. In order to increase the rate of smoking cessation of women smokers, research on women smokers should be given priority. It is important to identify socio-demographic characteristics known to be associated with smoking and smoking behavior, and to describe smoking-related behaviors and smoking cessation behaviors (Park, 2014). 

The intention to quit smoking is a prerequisite for preparation and implementation. In this study, the factors related to the intention of quitting tobacco use will be investigated by the multinomial logistic and multinomial probit regression methods using the micro data set of the Global Adult Tobacco Survey (GATS) conducted by Turkish Statistical Institute in 2008 and 2012. Since tobacco use is a preventable problem that causes deaths, effective policies can be developed to increase tobacco quitting by determining the factors that affect the intention of quitting tobacco use. 

The main purpose of this study was to examine and identify factors that affect the intention of women using tobacco in Turkey to stop tobacco use.

## Materials and Methods

*Data and Sample*

The microdata sets of “Global Adult Tobacco Survey (GATS)” conducted by the Turkey Statistical Institute for people ages 15 and above in 2008 and 2012 were employed in the study. GATS, which provides the opportunity to obtain key findings of the effects of tobacco control methods in 15 years and older adults, was carried out firstly in 2008 and in 2012 in the manner of household research representing the country. 

*Measures and Variables*

The dependent variable of the study is the intention of quitting tobacco use of women using tobacco measured by the question of “what is your opinion about quitting tobacco use (next month, after the next month and not intending to quit)”.

Independent variables that may have an impact on the intention to quit tobacco use are survey year (2008, 2012), age (15-24, 25-44, 45+), place of residence (rural, urban), educational status (not having finished a school, primary school, secondary school, high school, university), employment status (not working (student, housewife, retired, unemployed), working (paid/salaried/ self-employed)), car ownership (yes, no), the average number of cigarettes smoked per day (10 and fewer, 11-20 pieces, 21-30 pieces, 31 and more), first tobacco use (how long after waking up) (within the first 5 minutes, within 5-30 minutes, within 31-60 minutes, 61 minutes and later), attempt to quit tobacco use in the past year (yes, no), the frequency of tobacco use at home (daily, weekly, monthly/less than once a month, never), having information about whether tobacco use causes serious illness (yes, no ), awareness of anti-smoking messages (yes, no), awareness of health warnings on cigarette packages (yes, no) and exposure to stimulants that promote smoking (yes, no). Table 1 shows the independent variables used in the study and the frequencies and percentages according to women’s intention to quit tobacco use. 

*Statistical analysis*

In the study, statistical analyzes were performed using STATA 14 and SPSS programs. Firstly, the frequency and percentages of the women participating in the research were obtained according to their intention to quit tobacco use. Then, the chi-square tests of independence were performed to determine if there exists an association between women’s intention to quit tobacco use and socio-demographic and economic factors. Finally, factors affecting women’s intention to quit tobacco use and the impact dimensions of these factors were determined by employing multinomial probit regression analysis.

## Results

*Sample selection process*

In 2008 and 2012, 1,248 women out of 18,881 people, whose information was collected using the GATR, were included in the study. The focus of the study was women and therefore men (n = 4,269 + 4,470 = 8,739) were excluded from the analysis. In addition, those who did not smoke at the time of the survey (n=4,096+4,751=8,847) were not included in the study, since they had to be a tobacco user prior to having an intention to quit smoking. Therefore, as of the survey period, 1,295 women using tobacco were included in the evaluation. Some of the participants responded as “I don’t know” to the question “What is your opinion on quitting tobacco use?”, so they were also excluded from the study and finally, the data of 1,248 women were included in the model.

*Characteristic properties of the sample*

Table 1 shows the independent variables used in the study and the frequencies and percentages according to women’s intention to quit tobacco use.

Table 1 shows that 44% of women who have the intention of quitting tobacco use next month were present in the 2008 survey, while 56% of them were present in the 2012 survey. 11.3% of women who have the intention of quitting tobacco use next month were in the 15-24 age group, 60.1% were in the 25-44 age group and 28.6% were in the 45 and over age group. 43.5% of women who have the intention of quitting tobacco use next month were primary school and 10.7% were university graduates. 69% of women who have the intention of quitting tobacco use next month lived in urban areas, 77.4% of them did not work in any job and 42.9% of them owned a car.

*Chi-square tests of independence*

Before the multinomial probit model was established, the chi-square test of independence was performed and the results are given in Table 2. As seen in Table 2, it has been detect that there was a significant relationship between women’s intention to quit tobacco use and the variables of survey year, the average number of cigarettes smoked per day, first tobacco use (how long after waking up), attempt to quit tobacco use in the last one year, frequency of tobacco use at home, awareness of anti-smoking messages, awareness of health warnings on cigarette packages and exposure to stimulants that promote smoking.

*Estimation of model*

A multinomial probit regression model was used to determine the factors affecting the intention to quit tobacco use of the women aged 15 and over included in the study. VIF (Variance Inflation Factors) values among the independent variables to be included in the model were examined to test whether there is multicollinearity between the independent variables to be included in the multinomial probit regression analysis. It has been stated that variables with a VIF value of 5 and above cause moderate a multicollinearity problem, and those with a VIF value of 10 and above cause a high multicollinearity problem (Alkan and Abar, 2020). As seen in Table 3, no independent variable included in the model had a VIF value of 5 or greater. This indicates that there was no multicollinearity problem between the variables in the model.

The marginal effects of independent variables on the results of the estimated multinomial probit regression model and on the intention to quit tobacco use are presented in Table 3. 

According to Table 3, the probability of intention to quit tobacco use next month of women who participated in the survey in 2012 was 42.7% more than the reference group. The probability of intention to quit tobacco use next month of women who use their first tobacco within 31-60 minutes after waking up was 87.1% less than the reference group. The probability of intention to quit tobacco use next month of women trying to quit tobacco use in the last one year was 166.3% more than the others. The probability of intention to quit tobacco use next month of women who use daily tobacco at their home was 52.2% less than the reference group. The probability of intention to quit tobacco use next month of women who use daily tobacco at their home was 91.3% less than the reference group. The probability of intention to quit tobacco use next month of women who are aware of health warnings on cigarette packages was 110.8% less than the others.

The probability of intention to quit tobacco use after the next month of women who use their first tobacco within 31-60 minutes after waking up was 27.9% more than the reference group. The probability of intention to quit tobacco use after the next month of women trying to quit tobacco use in the last year was 28.4% more than the others. The probability of intention to quit tobacco use after the next month of women who are aware of health warnings on cigarette packages was 36.4% more than the others. The probability of intention to quit tobacco use after the next month of women who are exposed to stimulants that promote smoking was 27% more than the others.

**Table 1:** Distribution of Risk Factors Affecting Women Smokers’ Intention to Quit Tobacco Use

Variables		Intention to Quit Tobacco			Total
		Next Month	After Next Month	Not interested in quitting	
Survey year	2008	74 (44.0%)	281 (49.8%)	278 (53.9%)	633 (50.7%)
	2012	94 (56.0%)	283 (50.2%)	238 (46.1%)	615 (49.3%)
Age	15-24	19 (11.3%)	55 (9.8%)	61 (11.8%)	135 (10.8%)
	25-44	101 (60.1%)	359 (63.7%)	309 (59.9%)	769 (61.6%)
	45+	48 (28.6%)	150 (26.6%)	146 (28.3%)	344 (27.6%)
Place of residence	Rural	52 (31.0%)	158 (28.0%)	125 (24.2%)	335 (26.8%)
	Urban	116 (69.0%)	406 (72.0%)	391 (75.8%)	913 (73.2%)
Educational status	Not having finished a school	20 (11.9%)	56 (9.9%)	69 (13.4%)	145 (11.6%)
	Primary School	73 (43.5%)	235 (41.7%)	196 (38.0%)	504 (40.4%)
	Second School	23 (13.7%)	77 (13.7%)	72 (14.0%)	172 (13.8%)
	High School	34 (20.2%)	134 (23.8%)	116 (22.5%)	284 (22.8%)
	University	18 (10.7%)	62 (11.0%)	63 (12.2%)	143 (11.5%)
Employment status	Not working	130 (77.4%)	435 (77.1%)	371 (71.9%)	936 (75.0%)
	Working	38 (22.6%)	129 (22.9%)	145 (28.1%)	312 (25.0%)
Car ownership	Yes	72 (42.9%)	196 (34.8%)	188 (36.4%)	456 (36.5%)
	No	96 (57.1%)	368 (65.2%)	328 (63.6%)	792 (63.5%)
Average number of cigarettes smoked per day	10 and fewer	124 (73.8%)	378 (67.0%)	336 (65.1%)	838 (67.1%)
	11-20 pieces	36 (21.4%)	160 (28.4%)	146 (28.3%)	342 (27.4%)
	21-30 pieces	5 (3.0%)	16 (2.8%)	11 (2.1%)	32 (2.6%)
	31 and more	3 (1.8%)	10 (1.8%)	23 (4.5%)	36 (2.9%)
First tobacco use (How long after waking up)	Within the first 5 minutes	23 (13.7%)	51 (9.0%)	67 (13.0%)	141 (11.3%)
	Within 5-30 minutes	29 (17.3%)	98 (17.4%)	96 (18.6%)	223 (17.9%)
	Within 31-60 minutes	19 (11.3%)	116 (20.6%)	86 (16.7%)	221 (17.7%)
	61 minutes and later	97 (57.7%)	299 (53.0%)	267 (51.7%)	663 (53.1%)
Attempt to quit Tobacco	Yes	130 (77.4%)	290 (51.4%)	118 (22.9%)	538 (43.1%)
	No	38 (22.6%)	274 (48.6%)	398 (77.1%)	710 (56.9%)
Frequency of tobacco use at home	Daily	105 (62.5%)	390 (69.1%)	379 (73.4%)	874 (70.0%)
	Weekly	6 (3.6%)	33 (5.9%)	26 (5.0%)	65 (5.2%)
	Less than once a month	8 (4.8%)	29 (5.1%)	16 (3.1%)	53 (4.2%)
	Never	49 (29.2%)	112 (19.9%)	95 (18.4%)	256 (20.5%)
Disease information	Yes	164 (97.6%)	549 (97.3%)	497 (96.3%)	1210 (97.0%)
	No	4 (2.4%)	15 (2.7%)	19 (3.7%)	38 (3.0%)
Awareness of anti-smoking messages	Yes	160 (95.2%)	541 (95.9%)	468 (90.7%)	1169 (93.7%)
	No	8 (4.8%)	23 (4.1%)	48 (9.3%)	79 (6.3%)
Awareness of health warnings on cigarette packages	Yes	154 (91.7%)	543 (96.3%)	484 (93.8%)	1181 (94.6%)
	No	14 (8.3%)	21 (3.7%)	32 (6.2%)	67 (5.4%)
Exposure to stimulants that promote smoking	Yes	13 (7.7%)	56 (9.9%)	29 (5.6%)	98 (7.9%)
	No	155 (92.3%)	508 (90.1%)	487 (94.4%)	1150 (92.1%)

**Table 2:** The Results of the Chi-Square Test of Independence Related to Factors Affecting the Intention to Quit Tobacco Use

Risk Factors	χ²	Likelihood ratio χ²	Degree of freedom
	(P-value)	(P-value)	
Survey year	5.230 (0.073)^c^	5.238 (0.073)^c^	2
Place of residence	3.640 (0.162)	3.633 (0.163)	2
Age	2.156 (0.707)	2.162 (0.706)	4
Educational status	5.315 (0.723)	5.340 (0.721)	8
Employment status	4.516 (0.105)	4.486 (0.106)	2
Car ownership	3.672 (0.159)	3.617 (0.164)	2
Average number of cigarettes smoked per day	12.154 (0.059)^c^	12.172 (0.058)^c^	6
First tobacco use (How long after waking up)	12.621 (0.049)^b^	13.130 (0.041)^b^	6
Attempt to quit Tobacco	182.535 (0.000)^a^	190.377 (0.000)^a^	2
Frequency of tobacco use at home	13.752 (0.033)^b^	13.301 (0.039)^b^	6
Disease information	1.244 (0.537)	1.233 (0.540)	2
Awareness of anti-smoking messages	13.209 (0.001)^a^	13.012 (0.001)^a^	2
Awareness of health warnings on cigarette packages	6.616 (0.037)^b^	6.530 (0.038)^b^	2
Exposure to stimulants that promote smoking	6.918 (0.031)^b^	7.040 (0.030)^b^	2

^a^p<0.01; ^b^p<0.05; ^c^p<0.10

**Table 3:** Results of Estimated Multinomial Probit Model

Variables		Coefficients				Marginal effects		VIF
		Next month		After next month		Next month	After next month	
		β	Std. Error	β	Std. Error	ME	ME	
Survey year (reference category: 2008)								
	2012	0.401b	0.156	0.188	0.123	0.427b	0.055	1.05
First tobacco use (reference category: within the first 5 minutes)								
	Within 5-30 minutes	-0.252	0.27	0.21	0.232	-0.47	0.201	2.13
	Within 31-60 minutes	-0.484^c^	0.287	0.282	0.226	-0.871^b^	0.279^c^	2.14
	61 minutes and later	-0.186	0.225	0.215	0.2	-0.381	0.193	2.8
Attempt to quit Tobacco (reference category: no)								
	Yes	1.738^a^	0.162	0.932^a^	0.126	1.663^a^	0.284^a^	1.01
Frequency of tobacco use at home (reference category: never)								
	Daily	-0.473^b^	0.19	-0.165	0.161	-0.522^b^	-0.021	1.42
	Weekly	-0.670^c^	0.395	-0.043	0.273	-0.913^c^	0.084	1.21
	Less than once a month	-0.125	0.379	0.335	0.325	-0.412	0.225	1.18
Awareness of anti-smoking messages (reference category: no)								
	Yes	0.235	0.322	0.547^b^	0.235	-0.022	0.364^b^	1.04
Awareness of health warnings on cigarette packages (reference category: no)								
	Yes	-0.884^a^	0.329	0.079	0.293	-1.108^a^	0.283	1.04
Exposure to stimulants that promote smoking (reference category: no)								
	Yes	0.168	0.267	0.498^b^	0.221	-0.147	0.270^b^	1.01

^a^p<0.01; ^b^p<0.05; ^c^p<0.10

**Figure 1:**
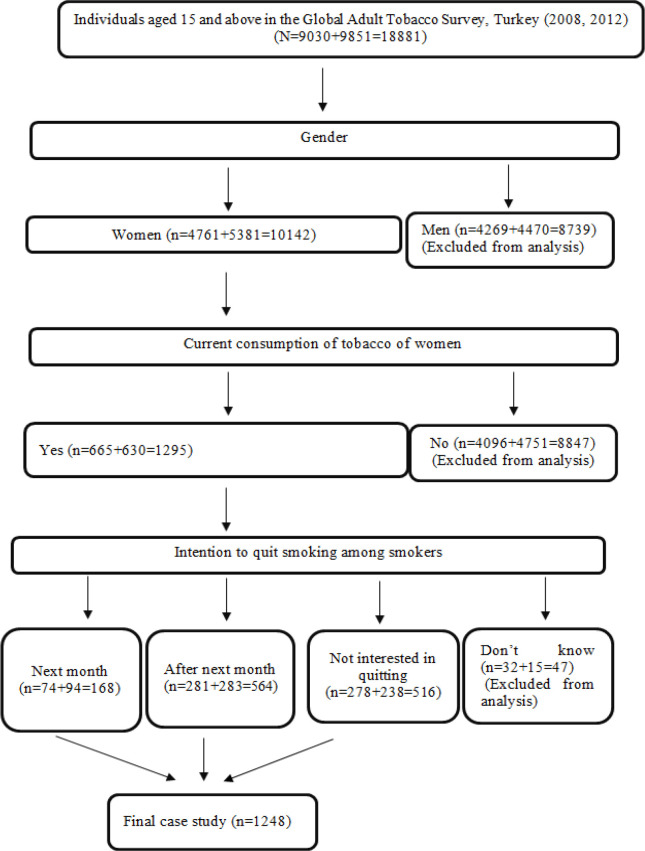
Selection Process of Women who Intend to Quit Smoking among Individuals from the GATS Survey

## Discussion

The results of the analysis showed that the variables of women’s first tobacco use after awakening up, survey year, attempt to quit tobacco use, frequency of using tobacco at home, awareness of anti-smoking messages, awareness of health warnings on cigarette packages and exposure to stimulants promoting smoking has an effect on the intention to quit tobacco use.

The study reveals that the probability of intention to quit tobacco use next month of women participating in the Global Adult Tobacco Survey in 2012 was more than that of women participating in 2008. It is obvious that it should be further combatted to quit tobacco use in order not to be faced with higher costs from health, social and economic aspects in the future.

In the study, attempt to quit tobacco use in the last 1 year was detected to be effective on the intention to quit tobacco use. The probability of intention to quit tobacco use of women who have tried to quit tobacco use in the last year was more than that of those who have not. Consistent with our results, there are also similar studies in the literature (Feng et al., 2010; Park, 2014; Wang and Mati, 2019). This can be considered as evidence that an attempt to quit smoking is a preliminary step in smoking cessation practice. These results suggest that smokers without a history of smoking cessation may not have any intention to quit smoking, and, therefore, short interventions can be planned to encourage motivation to quit smoking between this group (Feng et al., 2010). This finding shows that even if the first smoking cessation attempts are not successful, a smoker’s intention to quit will most likely remain high and subsequent smoking cessation attempts may prompt the user to the final successful cessation.

According to the frequency of tobacco use at home, the probability of intention to quit tobacco use of women whom tobacco was used daily and weekly at their home was found to be less than that of women who had never used tobacco at their home. Whether there is any constraint on tobacco use at home can influence individuals’ tobacco use. Similar literature studies have found that restrictions on smoking at home are associated with the intention to quit smoking (Myung et al., 2012; Owusu et al., 2017; Wang and Mati, 2019). Regarding restrictions on tobacco use, studies have reported that smokeless homes are associated with more attempts to quit smoking and with lower tobacco use and prevalence (Pizacani et al., 2004; Fong et al., 2006). Supporting the ban on tobacco use in common areas is related to the intention to quit smoking (Al-Zalabani et al., 2015). Therefore, the effort being made to prohibit tobacco use in closed spaces can also have an impact on the ban on tobacco use at homes. The fact that there is a restriction on the environment of tobacco users at home suggest that it will produce a positive result with the decrease in tobacco use rates.

This study revealed that awareness of anti-smoking messages has an effect on the intention to quit tobacco use. It has been concluded that the probability of intention to quit tobacco use of women being aware of the anti-smoking messages was more than that of those who be not. This result shows similarity with studies demonstrating that awareness of anti-smoking messages is related to the intention to quit smoking. (Caixeta et al., 2013; Wang and Mati, 2019). In addition, an association between exposure to anti-smoking messages in various places such as media, restaurant, public transportation, workplace, shop/store and the intention to quit tobacco use can be evaluated in parallel with the results of the study (Surani et al., 2012; Owusu et al., 2017). Mass media campaigns against tobacco use can help reduce the prevalence of tobacco use by discouraging individuals from tobacco use and encouraging existing users to quit their use. Anti-smoking information in mass media may be more effective when presented in multiple various places (Caixeta et al., 2013).

It has been determined that the probability of intention to quit tobacco use of women being aware of the anti-smoking messages was less than that of those who be not. It can be thought that there are no more impressive health warnings on cigarette packs or that the warnings are caused by a uniform or constant change. Unlike this result, literature results showed that exposure to picture warnings causes the increased awareness of smoking risks and increased interest in smoking cessation (Fathelrahman et al., 2010), and real pictures showing the destructive effects of smoking have a strong effect on the intention to quit smoking(Wu et al., 2015). 

An effective another variable in the study is the exposure to stimulants that promote smoking. Accordingly, the probability of intention to quit tobacco use of women exposed to stimulants (advertisement) that promote smoking is more than others. Similar findings were also obtained in the previous study (Emery et al., 2012). This may be associated with the reduction of the promotive efficiency of tobacco use due to the prevalence of anti-smoking messages laid down by the laws.

This study emphasizes that especially women, who smoke at their home, have no history of smoking cessation, are unaware of anti-smoking messages, are aware of health warnings on cigarette packs and use her first tobacco within 30-60 minutes after waking up, should be targeted. Further effort is needed to make at these groups’ intention to quit tobacco use, and programs to prevent and quit tobacco use should focus on these groups. 

It is important to understand the factors associated with the intention to quit that are decisive in quitting tobacco use. In contrast to many studies, the findings of this study will improve the limitation concerning the intention of women to quit tobacco use in the literature in Turkey. The results suggest that adopting tobacco bans at home can encourage users to quit. Strengthening tobacco use bans in confined spaces and providing information about the dangers of using these substances can help individuals to start thinking to quit using them. Furthermore, the findings could be a source of information in order to develop effective policies and programs to increase tobacco quitting rates. 
